# The Evaluation of a Rapid *In Situ* HIV Confirmation Test in a Programme with a High Failure Rate of the WHO HIV Two-Test Diagnostic Algorithm

**DOI:** 10.1371/journal.pone.0004351

**Published:** 2009-02-06

**Authors:** Derryck B. Klarkowski, Joseph M. Wazome, Kamalini M. Lokuge, Leslie Shanks, Clair F. Mills, Daniel P. O'Brien

**Affiliations:** Public Health Department, Médecins Sans Frontières, Amsterdam, The Netherlands; Columbia University, United States of America

## Abstract

**Background:**

Concerns about false-positive HIV results led to a review of testing procedures used in a Médecins Sans Frontières (MSF) HIV programme in Bukavu, eastern Democratic Republic of Congo. In addition to the WHO HIV rapid diagnostic test algorithm (RDT) (two positive RDTs alone for HIV diagnosis) used in voluntary counselling and testing (VCT) sites we evaluated *in situ* a practical field-based confirmation test against western blot WB. In addition, we aimed to determine the false-positive rate of the WHO two-test algorithm compared with our adapted protocol including confirmation testing, and whether weakly reactive compared with strongly reactive rapid test results were more likely to be false positives.

**Methodology/Principal Findings:**

2864 clients presenting to MSF VCT centres in Bukavu during January to May 2006 were tested using Determine HIV-1/2® and UniGold HIV® rapid tests in parallel by nurse counsellors. Plasma samples on 229 clients confirmed as double RDT positive by laboratory retesting were further tested using both WB and the Orgenics Immunocomb Combfirm® HIV confirmation test (OIC-HIV). Of these, 24 samples were negative or indeterminate by WB representing a false-positive rate of the WHO two-test algorithm of 10.5% (95%CI 6.6-15.2). 17 of the 229 samples were weakly positive on rapid testing and all were negative or indeterminate by WB. The false-positive rate fell to 3.3% (95%CI 1.3–6.7) when only strong-positive rapid test results were considered. Agreement between OIC-HIV and WB was 99.1% (95%CI 96.9–99.9%) with no false OIC-HIV positives if stringent criteria for positive OIC-HIV diagnoses were used.

**Conclusions:**

The WHO HIV two-test diagnostic algorithm produced an unacceptably high level of false-positive diagnoses in our setting, especially if results were weakly positive. The most probable causes of the false-positive results were serological cross-reactivity or non-specific immune reactivity. Our findings show that the OIC-HIV confirmation test is practical and effective in field contexts. We propose that all double-positive HIV RDT samples should undergo further testing to confirm HIV seropositivity until the accuracy of the RDT testing algorithm has been established at programme level.

## Introduction

Since 2000, Médecins Sans Frontières (MSF), in conjunction with the local Ministry of Health, has run a comprehensive HIV programme in Bukavu, a city located in the conflict affected eastern region of the Democratic Republic of Congo (DRC). Bukavu is home to significant numbers of people displaced by the conflict that has plagued the region since 1996. In 2004, the HIV prevalence in Bukavu was estimated to be 3.1%.[1]

Between January 2001 to May 2006, 13,678 clients were tested for HIV by nurse counsellors using rapid diagnostic tests (RDTs) performed according to the WHO HIV two-test algorithm [2].which interprets two positive independent tests as a positive result. There is no requirement for confirmation testing, which differs from the standard practice in developed countries, and there is no differentiation made between strongly and weakly positive test results. False-positive results with two independent HIV tests have been reported [3]–[11], but until recently this phenomenon was thought to be rare. It has also been assumed that confirmation testing in resource-limited settings was not feasible.

In late 2004, a group of patients from the MSF/MoH clinics who had initially tested positive for HIV on the basis of two positive RDT results were identified as having maintained a high CD4 count. They were retested with RDTs and shown to be HIV negative. The results were of great concern since a false-positive result may have significant adverse outcomes: stigma and discrimination, abandonment, domestic violence, exposure to unnecessary and potentially toxic medical treatment, and loss of confidence in the HIV programme. HIV counselling and testing practices were reviewed and supervision intensified; however the false-positive results persisted. The MSF algorithm was changed in December 2005 to introduce retesting of all VCT positive results and confirmation testing by Orgenics Immunocomb Combfirm HIV® (OIC-HIV) and/or western blot (WB). One of the study authors (JMW) observed that on visual reading of the rapid test result, some test lines were fainter than others raising the possibility that these weakly reactive tests may correlate with a higher risk of false-positive results.

In an observational study, we analysed 5 months′ routine programmatic data to compare the results obtained using the WHO 2-test algorithm with our new testing procedures under programme conditions. Our primary objective was to evaluate the performance of the OIC-HIV test compared to WB testing as a practical *in situ* confirmation test.

## Methods

### Study population

Testing was performed on consecutive VCT clients, drawn from both community health education activities and sexually transmitted infection clinics during January to May 2006. Programme details and treatment outcomes have been described elsewhere [12].

### Testing procedures

Clients were tested using Determine HIV-1/2® and UniGold HIV® rapid tests in parallel by nurse counsellors. The tests were performed in the presence of the client using plasma from EDTA venous blood samples collected and centrifuged immediately before testing. New gloves were used for each client. The specimen tube was labelled in front of the client using a unique client number. Manufacturers' instructions for test procedures were followed. Each test was interpreted by the counsellors as a positive result if both the test and internal control lines were positive. If both rapid tests were interpreted as positive, the client was informed that their result was positive but that a confirmation test would be done and that this might involve referral to an external laboratory. The individual was offered an immediate appointment in the HIV clinic, and accompanied to the clinic by the counsellor.

The counsellors further classified the results as strongly positive if the line had normal colour thickness and intensity or weakly positive if significantly thinner and weaker than normal positive results. If at least one of the two positive tests gave a weak result, the overall result was classified as weak positive.

The client was given a negative result if both tests were negative. If one test was positive and the other negative, the client was told the result was indeterminate and needed to be repeated in 6 weeks. All clients, regardless of the result, received routine counselling on risk reduction practices.

### Training and supervision of the nurse counsellors

Testing was done by the same four counsellors during the study period. All counsellors had programme experience and received training from the laboratory supervisor before the study. During the study period, the counsellors were directly observed performing the tests on a weekly basis by one of the study authors (JMW).

Daily supervision and psychosocial support was given to counsellors. Counsellors saw a maximum of eight clients per day to limit fatigue and ensure quality of testing and counselling.

### Quality control of VCT results

All double-positive results, whether weak or strong, were rechecked in the MSF reference laboratory using the same rapid test algorithm as that used in the VCT clinics. The laboratory control was performed unblinded on the same tube of plasma drawn by the counsellor. The laboratory technician further classified the results as strong or weak positives using the same criteria as the nurse counsellors. Samples from clients reported as negative by VCT were randomly selected for retesting in the laboratory.

### Quality control of test kits

All rapid test kits were stored according to manufacturers' instructions. Quality control was performed using a weak-positive sample prepared by the MSF reference laboratory on a randomly selected Determine and Unigold test each week and from every new box or pack.

### Confirmation testing

Plasma samples confirmed as double-positive on RDTs by the laboratory were tested using OIC-HIV and/or WB (GeneLabs Diagnostics HIV BLOT 2.2®). OIC-HIV was chosen as it requires no specialised laboratory equipment and thus can be easily performed in a peripheral laboratory, takes less than 2 hours, is easy to interpret, and separately detects p24, p31, gp40, gp120, and p36 antibodies. In addition, the HIV-OIC test uses recombinant and peptide antigens as do the RDTs and therefore can be expected to have similar sensitivity. WB was chosen as it is the internationally recognised gold standard.

WBs were performed at the National Reference Laboratory, Kigali, Rwanda. The laboratory was blind to the OIC-HIV results and the strength of rapid test reactivity. OIC-HIV tests were performed blind to WB results by the laboratory supervisor in the MSF referral laboratory.

The WB results were interpreted according to Centers for Disease Control criteria [13]. The result of the OIC-HIV test was interpreted according to both the manufacturer's directions (interpreting either 2 [gp120 and gp41] HIV envelope (*env*) antibodies or gp41 antibody plus 1 core antibody [p24 and/or p31] as a positive result) and according to a more stringent criterion requiring a minimum of two *env* (gp120 and gp41) antibodies plus one *gag* (p24) or *pol* (p31) reaction for a positive result.

### False positive diagnosis

This is defined as a sample that gives a positive test result with two independent RDT tests, and classified as positive according to the WHO two-test algorithm, which is not confirmed by WB testing.

### Statistical analysis

Analyses were performed using STATA Version 9 (STATACorp, Texas USA); 95% binomial exact CIs were used.

### Ethics review

Informed consent was obtained verbally from all clients before testing. The study was reviewed and approved by the MSF Ethics Review Board.

### Role of the funding source

There was no specific funding source. The study was funded as part of routine MSF operations.

## Results

### Study population

2864 clients received voluntary counselling and testing at the two test sites during the study period. Approximately 60% of the clients tested were female. The majority of clients (70%) reported residence in Bukavu city while the rest came largely from the rural areas surrounding the city. The most common reasons given for testing were ‘to know their status’, to confirm results received elsewhere, or a concern prompted by repeated illness, a sexually transmitted infection, or a history of sexual violence.

### VCT rapid test results

**Figure 1:**
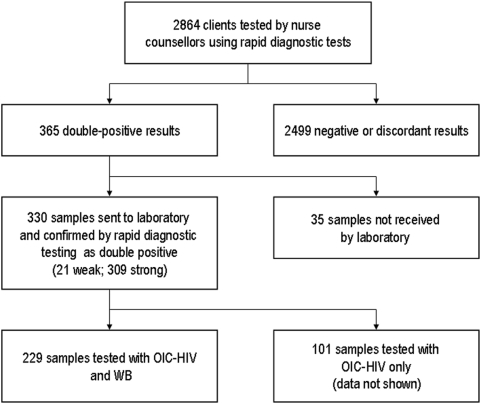
HIV Testing flowchart.

Figure 1 shows the flow of test results through the study. During January–May 2006, there were 365 (12.7%) double-positive results. Of these, 330 (90.4%) samples were referred to the laboratory. 35 samples were excluded because the counsellor was not able to draw a blood sample and therefore tested by finger prick, or because the sample did not arrive at the laboratory. Because of field constraints only 229 of the 330 samples were able to be referred for WB testing, the remainder were tested by OIC-HIV alone (data not shown). This paper only analyses the samples tested with both OIC-HIV and the gold standard reference WB methodology (Figure 1).

The laboratory confirmed all 60 negative VCT rapid test samples selected for quality control as negative.

### Laboratory rapid test, OIC-HIV, and western blot results

Repeat rapid tests were performed on 330 samples. There was 100% agreement between the VCT and laboratory rapid test results for positive/negative and strong/weak classification. All rapid test results presented here are laboratory results, not VCT results.

Table 1 shows that for the 229 samples tested by western blot, 205 were positive (203 had ≥5 HIV-1 bands) and 24 were negative or indeterminate, which gives an overall false-positive rate of 10.5% (95%CI 6.6–15.2). Of the 229 samples, 212 (92.6%) were strong, and 17 (7.4%) were weak, rapid test positives (Table 1). Seven of the strong positives were negative or indeterminate by WB, which gives a false-positive rate of 3.3% (95%CI 1.3–6.7). All 17 of the weak positives were negative or indeterminate by western blot, which gives a false-positive rate of 100% (95%CI 80.5–100). All 17 weak positives were also negative or indeterminate by OIC-HIV (Table 1).

**Table 1:** Analysis of double-positive rapid test results by enzyme immunoassay (OIC-HIV) and western blot (WB) banding pattern

Rapid test results (n = 229)	OIC-HIV reactions	WB banding pattern	n	OIC-HIV result	WB result^2^
**Strong rapid test positives; D+^s^ U+^s^ (n = 212)**	gp120+, gp41+, p24+, p31+	≥5 bands including gp120+, gp41+, p24+, p31+	199	POS	POS
	gp120+, gp41+, p24+ but p31−	≥5 bands including p31+	1	POS	POS
	gp120+, gp41+, p31+, p24+	≥5 bands but gp120−	3	POS	POS
	gp41+ p24+	gp41+ p24+	1	POS^3^	POS
	gp120+ gp41+	gp120+ p24+	1	POS^1^,^3^	POS
	**n confirmed POS by OIC-HIV/WB**		**205**		
	NEG	NEG	1	NEG	NEG
	NEG	p41+	2	NEG	IND
	p41+	NEG	1	IND	NEG
	gp41+ p24+	gp41+	1	POS^3^	IND
	gp120+ gp41+	gp41+	1	POS^3^	IND
	p24+ p31+	gp41+	1	IND	IND
	**n IND or NEG by OIC-HIV/WB**		7		
**Weak rapid test positives (n = 17)**					
**D+^s^U+^w^**	NEG	NEG	9	NEG	NEG
**D+^w^U+^s^**	NEG	gp41+	7	NEG	IND
**D+^w^U+^w^**	gp120+	NEG	1	IND	NEG
	**n IND or NEG by OIC-HIV/WB**		17		

D+^s^ =  Determine HIV-1/2® strong-positive. D+^w^ =  Determine HIV-1/2® weak-positive. U+^s^ =  UniGold HIV® strong-positive. U+^w^ =  UniGold HIV® weak-positive. POS = positive. NEG =  negative. IND =  indeterminate.

1 The discrepancy between banding patterns in OIC-HIV and WB may have been caused by either laboratory/clerical error, or different patterns of cross-reactivity between the two tests.

2 Using Centers for Disease Control criteria [13].

3 Using the manufacturer's interpretation.

### Agreement between OIC-HIV and western blot

Table 1 shows minor variations in banding patterns between OIC-HIV and WB in 19 of the 229 samples. Differences in cross-reactive patterns can be expected between WB whole viral antigens and OIC-HIV peptide/recombinant antigens [15]. The differences do not affect interpretation and are accepted as agreement.

Using the manufacturer's directions for interpretation of the OIC-HIV results, there was agreement between OIC-HIV and WB in classifying 227 of the 229 samples (99.1% [95%CI 96.9–99.9%]) as positive or negative/indeterminate (Table 1). For the two cases of disagreement, OIC-HIV gave a positive result whereas the WB was indeterminate (single gp41 band). This represents an over-diagnosis of 0.9%.

Using the more stringent criterion for OIC-HIV positivity, requiring a minimum of two *env* and one *gag* or *pol* reaction, there was agreement with the WB classification as positive or negative/indeterminate in 227 of the 229 samples (99.1% [95%CI 96.9–99.9%]). The OIC-HIV test gave no false positive results against WB. For the two cases of disagreement, OIC-HIV gave an indeterminate result whereas the WB was positive using CDC criteria [13]. In both cases, only two bands were detected by western blot.

## Discussion

Our findings demonstrate that two positive rapid HIV tests performed alone gave a 10.5% false positive rate compared to WB in our program in eastern DRC, and these findings strongly support the need for confirmation testing of double-positive HIV RDT test results. False-positive reactions in individual HIV serological tests have been widely reported for both RDT [6]–[9], [11], [16]–[19] and ELISA [3]–[5], [8], [10], [20]–[23] testing. A common practice in resource-limited countries, based on the WHO two-test algorithm [2], is to accept two positive HIV test results as diagnostic of HIV without performing confirmation testing. This will give an incorrect diagnosis if a false positive reaction occurs simultaneously in both tests, and this has been reported for a range of test brands and in varied locations [3]–[11]. The finding of a single gp41 reaction in 50% of our false positive cases is of particular concern because gp41 is shared by multiple RDT tests as a target antigen, and can therefore cause double false positive reactions.

Our study has the limitation that testing was performed under field conditions where it was not possible to follow up clients with negative or indeterminate WB results, or further confirm results by NAAT or p24-antigen testing. Therefore it is possible that some clients with positive RDT results and negative or indeterminate WB results may have been in early seroconversion. However a number of serological findings strongly support cross-reactivity as the major cause. The finding of a single gp41 band in 12/24 cases is not consistent with early phase infection and is more consistent with interference with non-HIV associated anti-gp41 as has been previously reported [22], [24], [25]. Further MSF field experience (data not shown) has demonstrated that Determine can detect lower levels of HIV antibody than Unigold, and therefore it is unlikely that the D+^w^U+^s^ and D+^w^U+^w^ results represent early seroconversion. It has also been reported that only a minority of indeterminate WB results subsequently seroconvert on follow-up testing [8], [24], [26]. None of the false-positive samples were from late-stage AIDS patients in whom reduced levels of *pol* and *gag* antibodies have been described.

The possibility that the false-positive results were a result of error in VCT is excluded because the results reported are based on the results obtained in the laboratory not during VCT. Although clerical error cannot be excluded it is unlikely given the high concordance between the results of the OIC-HIV testing performed in the MSF laboratory, and WB performed in a blinded manner in the Kigali laboratory. In addition, the false positives occurred using tests with different batch numbers, hence it is very unlikely that results were caused by test device error.

17 of the 24 samples that could not be confirmed by WB gave a weak reaction in one or both RDT tests. This is consistent with previously reported low specificity for weak reactivity in ELISA [5], [10], [20], [21] and RDT testing [11], [17], [19]. Therefore we propose that programmes that cannot perform confirmatory testing should repeat weakly reactive test results, and if confirmed interpret such reactions as an indeterminate rather than positive result, except in blood donor screening when a weak reaction should be considered as positive. However, and significantly, an unacceptably high 3.3% (95%CI: 1.3–6.7%) of double strong RDT positive samples also could not be confirmed by WB.

For reasons of practicality and cost, WB and molecular biology (NAAT) confirmation testing is not available in most resource-limited settings. For this reason we evaluated the OIC-HIV test as an *in situ* confirmatory test. This test is simple to use and interpret, has a relatively low cost compared with WB and NAAT, and requires no specialized laboratory equipment. Our findings show a 99.1% (95%CI 96.9–99.9%) correlation between OIC-HIV and WB (using the CDC minimum criteria [13]), with no false OIC-HIV positives if our proposed criteria were used, although there is a need for further research to confirm the reliability of this test in the African context.

However, using the manufacturer's recommended interpretation (2 *env*, or 1 *env* and 1 non-*env* reaction), the OIC-HIV test gave two false HIV1-positive results against WB. This may have been caused by gp41 activity; cross-reacting gp41 antibodies in HIV-negative sera have been reported to cause false gp120 and p24 reactivity [22], [25]. Until further data are available, we propose that 2 *env* and 1 *gag* or *pol* HIV-1 reaction, or 1 *env*, 1 *gag* and 1 *pol* should be the minimum requirement for a positive result. This should only delay diagnosis in a few cases of early seroconversion. In urgent situations, samples could be further tested by WB, p24 antigen assay, or NAAT techniques if available. However, the more stringent criterion should be used with caution in late stage AIDS, where p24 and p31 antibodies decline [27], or for screening blood donors. While there was close correlation between the reaction patterns of WB and OIC-HIV for the positive samples, there was considerable variability in the negative and indeterminate samples. We suggest the discordance was due to different reactivities of recombinant, peptide, and native viral antigens to cross-reacting antibodies [15], [28].

Our study design did not allow an analysis of factors causing cross reactivity, although several possibilities exist. Firstly, there is a high degree of HLA polymorphism among Africans [29], and because HLA class II antigens directly modulate the immune response [30], our population might have a heightened immune response to a commonly occurring infectious agent resulting in atypical frequency of cross-reactivity.

A further possibility is that non-specific polyclonal B-lymphocyte antibodies formed in any early immune response may interfere with HIV serological testing [15], [23], [31]. Such activity may be heightened in resource-limited settings by general immune activation due to multiple concomitant infections [32]–[34]. Gasaira *et al* [23] report that HIV false-positives were significantly more common in young children. The authors speculated that younger children have a less developed immune system against malaria and are therefore more likely to exhibit non-specific B-lymphocyte stimulation producing antibodies that cross-react with HIV antigens. Most of the WBs on false positive sera in their study showed gp41 and p51 reactivity. While most of our clients were adults, the study region has a recent history of population movement, and displaced persons are likely to have lower immunity to local diseases and be more likely to exhibit broad immune responses. Non-specific polyclonal antibody interference is consistent with our observed high frequency of weak positives and their low specificity. If such interference was a contributory factor to the false-positive rate, this would have implications for the testing of children, refugees, and internally displaced populations.

Our findings have important implications for scale-up of treatment in areas of low HIV prevalence. The PPV of the WHO two-test algorithm [2] is based on the assumption that each test performs independently. We found the PPV of the screening algorithm to be 89.6% at a prevalence of 11.4%; at an HIV prevalence of 5% and 2%, if the sensitivity and specificity of the tests is unchanged, the rapid-test-only protocol will give a PPV of 77.8% and 57.6% respectively. While it is impossible to determine the generalisability of our population, these results are concerning. We therefore propose that all double-positive HIV rapid test samples should undergo further testing to confirm HIV seropositivity until the accuracy of the testing algorithm has been established at the individual programme level. We have found this to be feasible in our settings using OIC-HIV. While rapid tests are an essential tool in efforts to make life-saving treatment available to those infected with HIV in resource-limited settings, the accuracy of rapid test algorithms, the low PPV of weakly reacting rapid tests, and alternatives to WB for confirmatory testing merit investigation.
